# Satellite glial cells drive the transition from acute to chronic pain in a rat model of hyperalgesic priming

**DOI:** 10.3389/fnmol.2023.1089162

**Published:** 2023-02-02

**Authors:** Junying Du, Min Yi, Danning Xi, Sisi Wang, Boyi Liu, Xiaomei Shao, Yi Liang, Xiaofen He, Jianqiao Fang, Junfan Fang

**Affiliations:** Key Laboratory of Acupuncture and Neurology of Zhejiang Province, Department of Neurobiology and Acupuncture Research, The Third Affiliated Hospital of Zhejiang Chinese Medical University, Zhejiang Chinese Medical University, Hangzhou, China

**Keywords:** neuroinflammation, pain transition, satellite glial cell, CXCL1, chemokine

## Abstract

Chronic pain is one of the most common clinical syndromes affecting patients’ quality of life. Regulating the transition from acute to chronic pain is a novel therapeutic strategy for chronic pain that presents a major clinical challenge. However, the mechanism underlying pain transitions remains poorly understood. A rat hyperalgesic priming (HP) model, which mimics pain transition, was established decades ago. Here, this HP model and RNA sequencing (RNA-seq) were used to study the potential role of neuroinflammation in pain transition. In this study, HP model rats developed prolonged hyperalgesia in the hind paw after carrageenan (Car) and PGE2 injection, accompanied by obvious satellite glial cell (SGC) activation in the dorsal root ganglion (DRG), as indicated by upregulation of GFAP. RNA-Seq identified a total of differentially expressed genes in the ipsilateral DRG in HP model rats. The expression of several representative genes was confirmed by real-time quantitative PCR (qPCR). Functional analysis of the differentially expressed genes indicated that genes related to the inflammatory and neuroinflammatory response showed the most significant changes in expression. We further found that the expression of the chemokine CXCL1 was significantly upregulated in the rat DRG. Pharmacological blockade of CXCL1 reduced protein kinase C epsilon overproduction as well as hyperalgesia in HP rats but did not prevent the upregulation of GFAP in the DRG. These results reveal that neuroinflammatory responses are involved in pain transition and may be the source of chronic pain. The chemokine CXCL1 in the DRG is a pivotal contributor to chronic pain and pain transition in HP model rats. Thus, our study provides a putative novel target for the development of effective therapeutics to prevent pain transition.

## 1. Introduction

Chronic pain is one of the most common clinical syndromes in humans and is characterized by plastic changes in the peripheral nervous system (PNS) and central nervous system (CNS), also called peripheral and central sensitization (Ji et al., 2018; Vincent, 2020). A vast number of functional proteins and signaling pathways have been reported to be altered or changed in the peripheral neurons in mammals with chronic pain (Meade and Garvey, 2022; Zheng et al., 2022). They are thought to contribute to chronic pain directly or indirectly and might be targets for pain treatment (Berta et al., 2017). Although many changes in peripheral sensory neurons related to chronic pain have been discovered, the trigger of these changes is still unknown.

Nearly a decade ago, a hyperalgesic priming (HP) animal model was first used to study how chronic pain in the initial phase is different from acute pain (Price et al., 2018; Glare et al., 2019). In this animal model, intraplantar PGE2 injection after recovery from acute inflammatory stimulation was found to produce long-lasting hyperalgesia that was significantly beyond its ability to lead to acute pain (Ferrari et al., 2013). Protein kinase C epsilon (PKCε) has been identified as the key molecule involved in this phenomenon, and its activation in dorsal root ganglion (DRG) neurons induces the transition from acute pain to chronic pain (Fang et al., 2021; Wang et al., 2021). Our group recently found that in this model, changes in the expression of some receptors on neurons, such as upregulation of mGluR5 and downregulation of GABAAR, are involved in the activation of PKCε in the DRG (Wang et al., 2020, 2021). However, it is still not clear whether non-neuronal cells in the PNS are involved in pain transition and PKCε activation.

In the PNS, the somata of sensory neurons are tightly wrapped by satellite glial cells (SGCs), thereby preventing synaptic contact (Hanani and Spray, 2020). Accumulating evidence indicates that SGC activation in sensory ganglia during or following nerve damage or peripheral inflammation contributes to pain (Ceruti, 2021). Activation of SGCs induces the release of bioactive substances, including ATP, glutamate, cytokines and chemokines such as tumor necrosis factor (TNF), IL-1β and CCL2 (Ji et al., 2019; Hanani and Spray, 2020). These substances act on peripheral nociceptive neurons to induce neuroinflammation and alter the function of pain-related ion channels or the expression level of receptors to initiate peripheral sensitization (Montague et al., 2018; Valdez-Morales et al., 2022; Xu et al., 2022). In addition, SGCs express receptors for substances released from peripheral neurons, most notably CX3CR1 and P2X7 receptors (Souza et al., 2013; Neves et al., 2020). These receptors in the PNS also contribute greatly to chronic pain (Silva and Malcangio, 2021; Hu et al., 2022). Furthermore, close contact permits bidirectional communication and functional interactions between sensory neurons and SGCs, including activation of neighboring neurons and SGCs. The discovery of crosstalk between SGCs and sensory neurons raises interesting questions, does SGC activation result in pain transition, does SGC activation result in PKCε activation, or does the increased PKCε expression induce the SGC reactivity?

To further explore the peripheral mechanism underlying pain transition, we carried out genome-wide expression profiling of ipsilateral DRG tissues from HP model rats and sham HP model rats using RNA sequencing (RNA-Seq). A number of differentially expressed genes were identified. The molecular and cellular functions of these genes and the signaling pathways in which they are involved were examined. We also compared our datasets with a previously published set of data from classical chronic pain models. By analyzing our findings, CXCL1 was identified as a key molecule in the DRG in the transition from acute to chronic pain. Our work revealed that SGCs drive the transition from acute to chronic pain in a rat model of HP, an essential finding for understanding pain transition mechanisms. Our findings may provide further insights for the identification of novel and effective therapeutic targets for chronic pain in the initial phase.

## 2. Methods and materials

### 2.1. Animals

Adult male Sprague Dawley (SD) rats [weighing 180–230 g) (animal certificate no. SCXK(Hu)2013–0016, Shanghai Laboratory Animal Center, Chinese Academy of Sciences] were used for this study. The animals were housed five per cage in the laboratory animal center of Zhejiang Chinese Medical University [SYXK(Zhe)2013–0184] in a controlled environment (25°C ± 2°C, 50% ± 10%) on a 12 h light/dark cycle with food and water provided *ad libitum*. All animal procedures performed in this study complied with institutional and governmental regulations regarding the ethical use of animals and were approved by the Experimental Animal Center affiliated with Zhejiang Chinese Medical University (approval no. IACUC-20180319-12).

### 2.2. Hyperalgesic priming model

Carrageenan (Car) and PGE2 were purchased from Sigma–Aldrich (St. Louis, MO, United States). A stock solution of PGE2 (1 μg/μl) was prepared in 10% ethanol and mixed with normal saline to a concentration of 100 ng/25 μl immediately before injection. Car was dissolved in normal saline to a concentration of 2% and stored.

Hyperalgesic priming was induced as previously described (Wang et al., 2021). Rats were briefly anesthetized, and HP was induced by intraplantar injection of 100 μl of Car (1st injection) followed by injection of 25 μl of PGE2 (2nd injection) 7 days after the 1st injection. In the sham HP group, the same volume of normal saline (NS, 0.9% NaCl) was administered instead of Car.

### 2.3. Behavior testing

Nociceptive behaviors were quantified before the 1st injection, 4 h and 7 days after the 1st injection, and 1 and 24 h after the 2nd injection. The experimenters were blinded to the study conditions for the duration of the experiment.

Paw withdrawal thresholds (PWTs) were tested using von Frey filaments (Stoelting, IL, United States) by the up–down method as previously described. Von Frey filaments (0.4, 0.6, 1, 2, 4, 6, 8, 15, and 26 g) were pressed onto the lateral plantar surface of the ipsilateral paw. The first filament applied corresponded to a force of 4 g. A filament with a higher or lower force was then chosen depending on whether the response was negative or positive. The responses were recorded as X or O. The results were calculated using a previously described function (Chaplan et al., 1994).

### 2.4. Tissue preparation for RNA-Seq

A total of five rats were included in the HP and sham HP groups. According to the experimental design, the animals were deeply anesthetized with isoflurane and perfused through the ascending aorta with normal saline at 24 h after PGE2 injection. After perfusion, the L4-L5 DRGs were harvested and stored at –80°C. Total RNA was extracted using TRIzol reagent, and the RNA concentration and purity were assayed using a NanoDrop 1,000 spectrophotometer. RNA integrity was checked by electrophoresis on a 2% (m/v) agarose gel. After these tests, six samples of sufficient quality (three samples from each group) were sent for microarray analysis.

### 2.5. RNA-Seq library establishment and RNA-Seq

Approximately 1 μg of total RNA per sample was treated with reagents from the Bibo-Zero TM Magnetic Kit to deplete rRNA. cDNA was synthesized by reverse transcription with random primers and was subjected to end-repair and 3′ adenylation. Adapters were ligated to the ends of these 3′ adenylated cDNA fragments. To enrich the cDNA fragments, multiple rounds of PCR amplification were performed with PCR Primer Cocktail and PCR Master Mix. Finally, the PCR products were purified with AMPure XP Beads and were quality controlled and quantified by two methods: checking the distribution of the fragment size using an Agilent 2,100 Bioanalyzer and quantifying the library using real-time quantitative PCR (qPCR) (TaqMan Probe). The qualified libraries were subjected to paired end sequencing on the HISeq 4,000 platform (Beijing Genomic Institute, Shenzhen, China).

### 2.6. Bioinformatics analysis

The raw reads (primary sequencing data) were subjected to quality control. Information on the total reads and mapping ratio of the reads is shown in Table 1. Bioinformatics analysis was performed as previously described (Chen et al., 2020). In brief, reads containing sequencing adapters, those with a high rate of low-quality bases, and those with an unknown base rate of greater than 5% were removed by SOAPnuke (v1.5.2). Then, the clean reads were saved in FASTQ format. Afterward, HISAT2 (v2.0.4) was used to map the clean reads to the reference genome. Then, Ericscript (v0.5.5) and rMATS (v3.2.5) were used to detect fusion genes and alternatively spliced genes, respectively. Bowtie2 (v2.2.5) was applied to align the clean reads to the gene set, a database built by BGI, in which known and novel coding transcripts were included. Then, the expression levels of genes were calculated by RSEM (v1.2.12). The whole bioinformatics analysis process was carried out by BGI.

**Table 1:** Information on total and clean reads.

	Total raw reads (*M*)	Total clean reads (*M*)	Clean reads ratio (%)	Total mapping (%)
Sham HP-1 (NS + PGE2)	45.57	44.57	97.79	94.54
Sham HP-2	45.57	44.51	97.67	94.74
Sham HP-3	45.57	45.14	99.05	95.06
HP-1 (Car+PGE2)	45.57	43.93	96.40	94.02
HP-2	45.57	44.30	67.40	94.44
HP-3	45.57	44.44	97.52	94.75

### 2.7. Cluster analysis and screening of differentially expressed genes

Significantly differentially expressed mRNAs (DeRNAs) were identified through volcano plot filtering. The threshold used to screen for up-and downregulated mRNAs was |log2(fold-change)| > 1.2 (*p* < 0.05). Hierarchical clustering as well as heatmap generation was carried out by using the “pheatmap” package in Bioconductor R software. The DeRNAs were further subjected to pathway (KEGG) and gene ontology (GO) enrichment analyses using Dr. Tom (BGI). For GO enrichment analysis, the genes were annotated and classified according to the biological process (BP), cellular component (CC), and molecular function (MF) terms in which they were enriched. For KEGG pathway analysis, pathways were ranked by their enrichment score.

### 2.8. Protein–protein interaction network analysis

Protein–protein interaction (PPI) network analysis was performed as previously described (Chen et al., 2020). In brief, Search Tool for the Retrieval of Interacting Genes (STRING) was used to obtain information regarding predicted and experimentally determined protein interactions. The predictions made by this database are derived from neighborhood, gene fusion, cooccurrence, and coexpression analyses; databases; and text mining. The web-based STRING database was used to predict PPIs after the list of overlapping genes was uploaded *via* the search bar. Based on the interactions, a PPI network was established and then visualized using Cytoscape software.

### 2.9. Source of microarray data

Two independent datasets from neuropathic pain and inflammatory pain models, namely, the spared nerve injury (SNI) and complete Freund’s adjuvant (CFA) models, were selected for this study. The SNI (GSE30691) and CFA (GSE24431) datasets were downloaded from Gene Expression Omnibus (GEO) at https://www.ncbi.nlm.nih.gov/geo/. Genes in the CFA model with *p* < 0.05 and an absolute value of fold change >1.5 were identified as differentially expressed genes according to the original manuscript (Chang et al., 2010). Genes in SNI model with *p* ≤ 0.01 and an absolute log2(fold change) >1.25 were identified as differentially expressed genes according to the original manuscript (Costigan et al., 2010).

### 2.10. DRG injection

Drugs were directly injected into the DRG as described in a previous report (Ferrari et al., 2007). Briefly, the rats were anesthetized by the inhalation of 2% (v/v) isoflurane. A G30 needle was inserted into the intervertebral space between the 5th and 6th lumbar vertebrae. The needle was considered to have reached the space when the resistance of the bone was diminished, and a paw flinch reflex was observed.

### 2.11. Real-time quantitative PCR

Total RNA extracted from L4-L5 DRG tissues was reverse transcribed into cDNA using random hexamer primers. Relative mRNA levels were quantified by RT–PCR using the fluorescent EvaGreen dye method. cDNA was subjected to qPCR using the CFX96™ Real-Time PCR Detection System (Bio-Rad, United States). The sequences of all primers used are shown in Table 2. GAPDH was used as an internal reference gene. Each sample was measured in triplicate, and data points were examined for integrity by analysis of the amplification plot. Relative RNA levels were calculated by the 2^-ΔΔCt^ method.

**Table 2:** Primer sequences.

	Primer(5′-3′)
GAPDH	CTGGAGAAACCTGCCAAGTATG
	GGTGGAAGAATGGGAGTTGCT
Ca9	CACTTGGAAGAAATCGCAGAGG
	GGAAACGGAGAGCGTATGGA
Mn1	TCTGCTTCACGGACCTTCAT
	AGCGGATACTTCAGCCTCTTCT
Rpl30	GTCAATCAACTCTCGGCTCCAA
	ACTCTGTAGTATTTTCCACACGCTG
Phex	GGGGTTTATCCTTGGCTGAGA
	CGGAATGTTGCCAGTGTTTGC
Mdh1b	GAGGAGTGGCTGGAAGATGTC
	GTATCCTCCCAAAAGCAAGCC
CXCL1	GCCACACTCAAGAATGGTCG
	GACGCCATCGGTGCAATCTA
IL-1β	GCACAGTTCCCCAACTGGTA
	ACACGGGTTCCATGGTGAAG
TNF-α	ACCATGAGCACGGAAAGCAT
	AACTGATGAGAGGGAGCCCA

### 2.12. Immunofluorescence

DRGs were sliced at a thickness of 12 μm. The slices were blocked with 5% normal donkey serum in TBST (1% Tween 20) for 1 h at 37°C and then incubated with a rabbit anti-GFAP antibody (1:1000 in 5% normal donkey serum, Abcam, United States) overnight at 4°C. The slices were then incubated in a fluorescein AffiniPure donkey anti-rabbit IgG (Alexa 488-conjugated, Abcam, United States) for 1 h at 37°C. Images of GFAP expression in the L4 and L5 DRGs were acquired by using an Imager M2 (Zeiss, Germany). Neurons in which more than 50 of their circumference was surrounded by GFAP-positive SGCs were counted, and the data are expressed as the percentage relative to the total number of neurons present in the field (Warwick and Hanani, 2013).

### 2.13. Western blotting

The method used for western blotting was described previously. Briefly, total protein was extracted from L4-L5 DRGs tissues. RIPA lysis buffer (Beyotime, China) containing 1% PMSF (Beyotime, China) and a protease/phosphatase inhibitor cocktail (Applygen, China) was used to extract protein. The protein concentration was measured by a BCA protein assay kit. Protein samples (20 μg) were separated on 5% SDS–PAGE gels and electrophoretically transferred to polyvinyl difluoride (PVDF) membranes (Bio-Rad, United States). The membranes were incubated with 5% low-fat milk in TBST for 1 h at room temperature, with rabbit anti-PKCε (1:1000 in 5% normal goat serum, Abcam, United States) and anti-GFAP (1:1000 in 5% normal goat serum, Abcam, United States) overnight at 4°C, and with horseradish peroxidase (HRP)-conjugated goat anti-rabbit IgG (1:5000, Abcam, United States) for 1 h at room temperature. A rabbit anti-GAPDH (HRP conjugate) (1:1000, CST, United States) antibody was used as the internal control. The membranes were visualized with an ECL kit (Pierce, United States), and the signals were acquired with the ImageQuant LAS 4000 system (EG, United States). The density of each band was measured using ImageQuant TL 7.0 analysis software (GE, United States). The mean expression level of the target proteins in animals from the 1st group was considered to be 1, and the relative expression levels of the target proteins in all animals were normalized to the level in the 1st group.

### 2.14. Statistical analysis

The data in the graphs are presented as the means ± SEMs. Student’s *t-*test was used to compare two independent samples, whereas analysis of variance (ANOVA) followed by Bonferroni’s multiple comparison tests was used to compare three or more samples. *p* < 0.05 was considered statistically significant.

## 3. Results

### 3.1. Satellite glial cell reactivity was induced by Car/PGE2 injection and involved in the chronic phase of pain in the HP model

**Figure 1:**
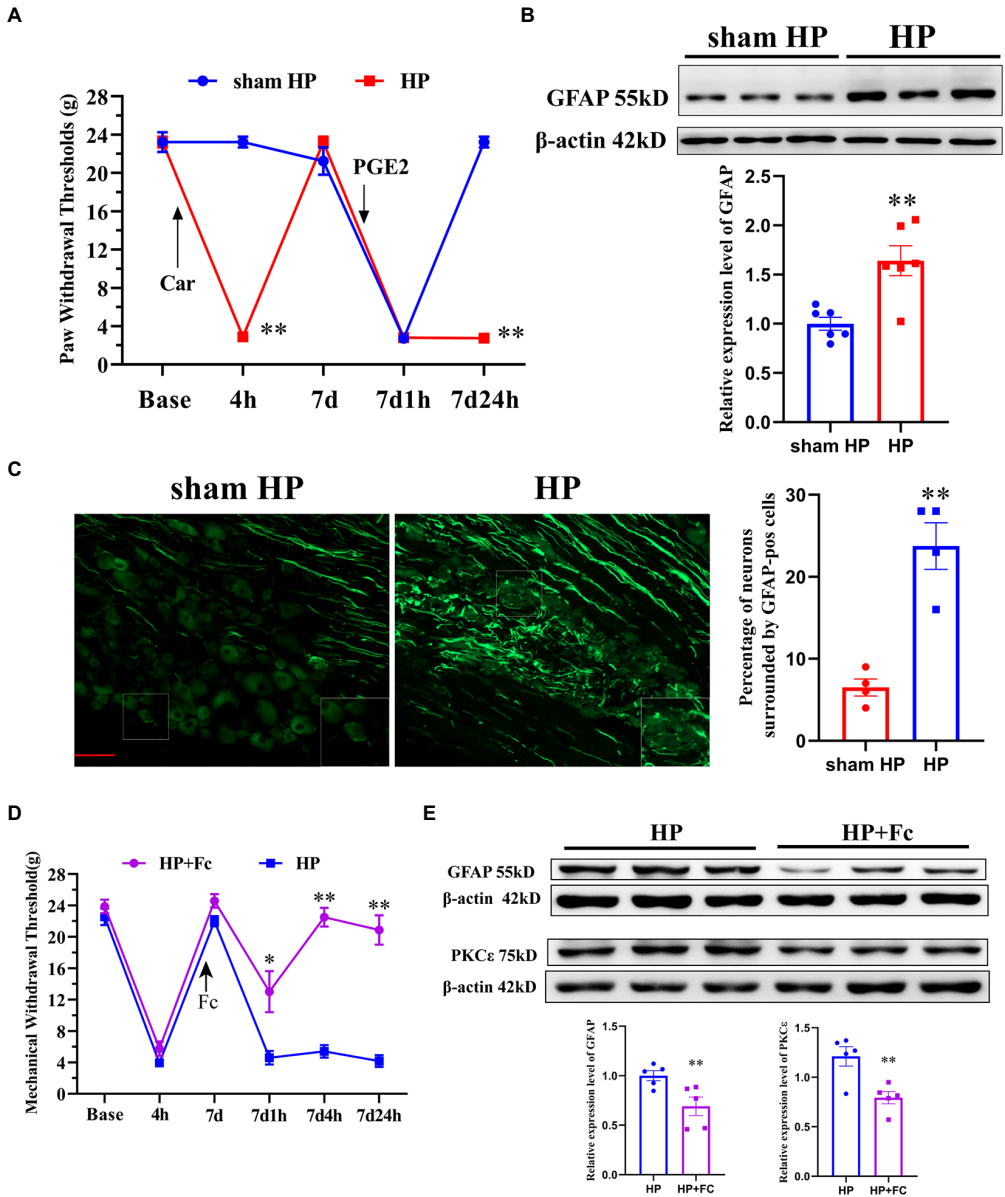
Induction of SGC reactivity plays a key role in the initiation of pain transition. **(A)** The mechanical withdrawal thresholds (MWTs) of rats that received Car (2%, 100 μl/paw) and PGE2 (100 ng/25 μl/paw) injections. *n* = 5 rats per group. **(B)** Quantification of the Western blot results and a representative Western blot showing GFAP protein expression levels in the DRG 24 h after PGE2 injection. **(C)** Representative images of GFAP-positive SGCs (the enlarged image is in the lower right corner) in the lumbar DRGs of HP model rats. Quantification of neurons surrounded by GFAP-positive SGCs the in L4–L5 DRGs. **(D)** Injection of Fc (an antagonist of SGC activation, 2 nmol, three times) into the L5 DRG before PGE2 injection produced analgesic effects in HP model rats. *n* = 6 rats per group. **(E)** Quantification of the Western blot results and a representative Western blot showing GFAP and PKCε protein expression levels in the DRG 24 h after PGE2 injection. *p < 0.05, **p < 0.01.

Car injection followed by PGE2 injection was used to establish a classical animal model called the HP model, which can be used to study the transition from acute to chronic pain (Ferrari et al., 2015). Intraplantar Car/PGE2 injection into the left hindpaw resulted in long-lasting mechanical hyperalgesia (Figure 1A) and significantly upregulated GFAP expression in the DRG (Figure 1B) in SD rats, suggesting the induction of SGC reactivity. Furthermore, the percentage of neurons surrounded by GFAP-positive cells in the DRG was significantly increased (Figure 1C). Collectively, these results indicated that SGCs reactivity in the ipsilateral DRG was induced by Car/PGE2 injection. To determine whether SCGs are necessary for Car/PGE2-induced long-lasting pain, we examined the effect of fluorocitrate (Fc) on HP model rats. Prevention of SGC reactivity by injection of Fc (2 nmol, three times) into the ipsilateral L5 DRG before PGE2 injection significantly attenuated Car/PGE2-induced long-lasting hyperalgesia (Figure 1D) and downregulated the expression of GFAP in the lumbar DRG (Figure 1E). Previous studies have demonstrated that PKCε activation in the DRG is a molecular marker of pain transition initiation (Wang et al., 2021). Our previous studies also showed that inhibition of high PKCε expression reverses chronic pain in HP model rats (Fang et al., 2021). Here, we tested whether blocking the induction of SGC reactivity affects PKCε expression in the L5 DRG. Injection of Fc into the DRG before intraplantar PGE2 injection significantly downregulated the expression of GFAP in the DRG (Figure 1E), which indicated that PKCε activation in the DRG might be a response to SGC reactivity.

### 3.2. Overview of differential mRNA expression throughout the transition from acute to chronic pain

Because a few cytokines and chemokines are produced by SGCs, we further carried out genome-wide expression profiling of ipsilateral DRG tissues from HP and sham HP model rats using RNA-Seq (24 h after PGE2 injection).

**Figure 2:**
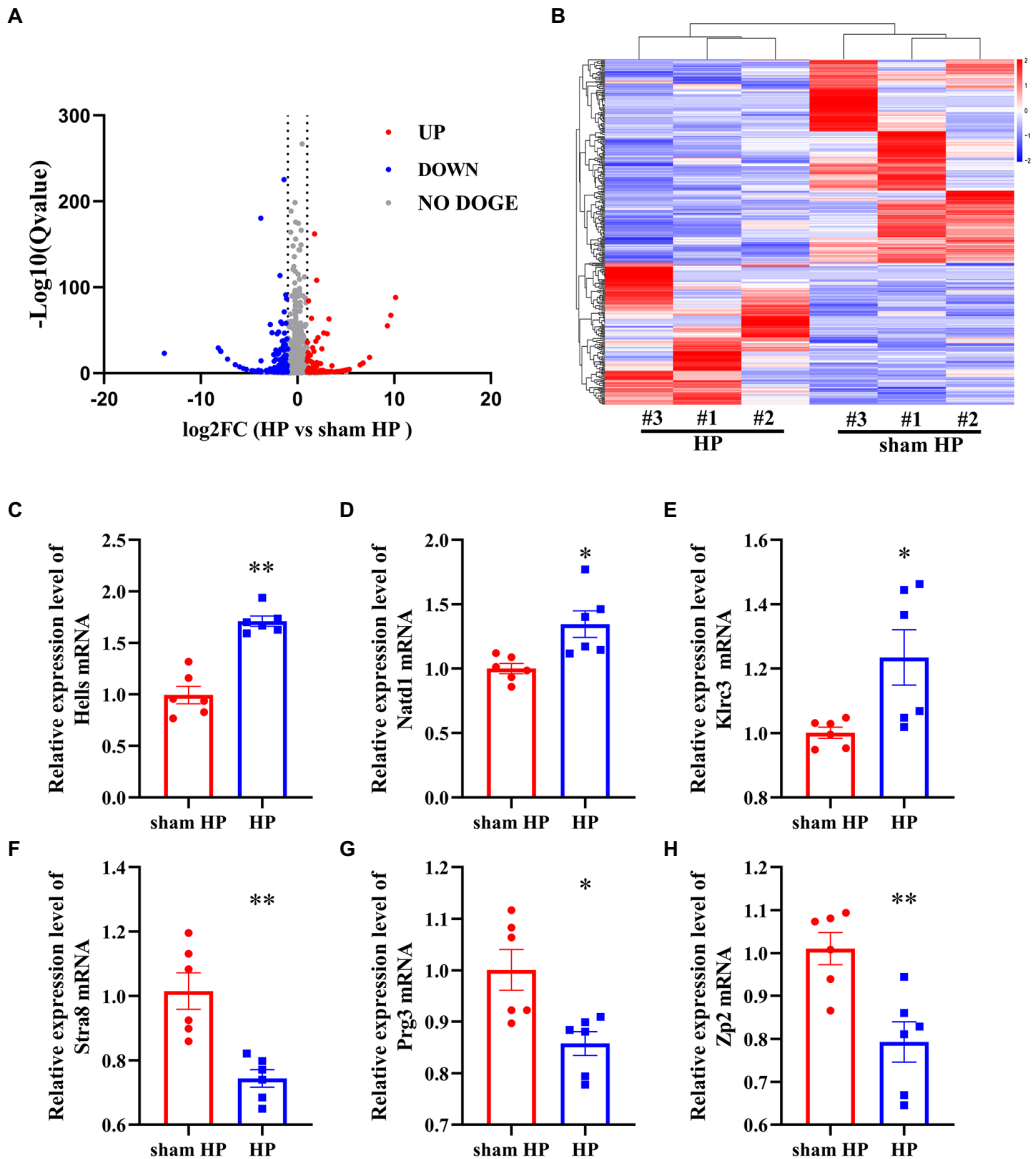
RNA-Seq reveals gene expression changes in the DRG induced by HP. **(A)** Volcano plot showing gene expression profiles in the ipsilateral DRG in the HP group compared with the sham group. Red and blue spots indicate up-and downregulated DeRNAs, respectively, whereas gray spots indicate non-DEGs. **(B)** Heatmap displaying the hierarchical clustering of DeRNAs in the HP and sham HP groups. **(C–H)** Validation of the RNA-Seq results *via* qPCR. The expression of six randomly selected DeRNAs identified by RNA-Seq was examined by qPCR. *n* = 6 rats per group. *p < 0.05, **p < 0.01.

The DeRNAs involved in the transition from acute to chronic pain between the HP model and sham HP model rats were analyzed 24 h after PGE2 injection. The results showed that 355 mRNAs, 146 of which were upregulated and 209 of which were downregulated, were significantly differentially expressed in the HP group, compared with the sham HP group (Figure 2A). The most upregulated mRNAs were LOC103693999, LOC103690175, Chchd2, RGD1564887, and Rpl30 (Table 3). The most downregulated mRNAs were LOC108348064, LOC679565, Kdm4d, Stra8, and LOC108348079 (Table 4). Hierarchical cluster analysis of the DeRNAs showed that the three sham HP samples were clustered together, the three HP samples were clustered together, and the results were highly consistent (Figure 2B).

**Table 3:** The top 20 upregulated mRNAs.

Upregulated gene	Gene ID	Location	Log2 fold change (HP/sham HP)	*Q* value	Official gene name (NCBI)
LOC103693999	103693999	81,549,184–81,583,637	10.15	9.7E-89	Transmembrane and coiled-coil domain-containing protein 3
LOC103690175	103690175	158,265,270–158,288,228	9.66	5.1E-68	PHD finger protein 6
Chchd2	316643	94,500,439–94,501,110	9.31	9.4E-56	Coiled-coil-helix-coiled-coil-helix domain containing 2
RGD1564887	499363	262,861,241–262,891,231	7.46	3.8E-19	Similar to 9130011E15Rik protein
Rpl30	64640	65,648,470–65,651,363	6.82	0	Ribosomal protein L30
LOC103692976	103692976	140,149,991–140,172,615	6.79	1.10E-12	Cyclin-T1-like
LOC100912471	100912471	14,760,054–14,830,055	6.73	3.20E-12	Cytochrome P450 4F5-like
LOC102551539	102551539	10,497,708–10,874,111	6.47	1.90E-10	Zinc finger protein 709-like
LOC108348106	108348106	90,957,132–90,962,541	5.36	3.28E-05	Alpha-ketoglutarate-dependent dioxygenase alkB homolog 6
LOC103689943	103689943	28,267–72,912	5.18	1.12E-04	Meiosis arrest female protein 1-like
LOC103689934	103689934	109,901,916–109,903,783	5.18	1.18E-04	Leucine-rich repeat-containing protein 45-like
Car9	313495	59,008,277–59,015,535	4.95	4.68E-04	Carbonic anhydrase 9
LOC100912478	100912478	35,126,955–35,134,495	4.85	8.08E-04	Vacuolar ATPase assembly integral membrane protein VMA21-like
LOC690948	690948	63,963,855–63,971,869	4.8	1.04E-03	Similar to paired-Ig-like receptor A11
Tmem45a	680866	46,714,401–46,779,503	4.68	1.87E-03	Transmembrane protein 45A
LOC103692307	103692307	18,442,950–18,446,389	4.67	2.04E-03	Protein SON-like
LOC100910308	100910308	33,563,887–33,977,623	4.65	2.17E-03	Multifunctional protein ADE2-like
Gsta1	24421	27,364,746–27,381,004	4.54	3.65E-03	Glutathione S-transferase alpha 1
LOC102555324	102555324	14,358,036–14,359,895	4.42	6.01E-03	MLV-related proviral Env polyprotein-like
Ugt1a1	24861	95,295,701–95,302,822	4.3	9.25E-03	UDP glucuronosyltransferase family 1 member A1

**Table 4:** The top 20 downregulated mRNAs.

Downregulated gene	Gene ID	Location	Log2 fold change (HP/sham HP)	*Q* value	Official gene name (NCBI)
LOC108348064	108348064	72,608,217–72,629,257	−13.77	8.20E-24	Gamma-secretase subunit APH-1B-like
LOC679565	679565	845,234–848,196	−8.18	3.70E-30	Similar to acyl-Coenzyme A binding domain containing 5
Kdm4d	689582	12,968,192–12,993,651	−7.97	1.30E-26	Lysine demethylase 4D
Stra8	500079	62,447,724–62,474,802	−7.93	4.1E-26	Stimulated by retinoic acid 8
LOC108348079	108348079	124,516,931–124,518,080	−7.22	4.7E-17	RING finger protein 113A-like
LOC100362684	100362684	161,889,229–161,889,746	−6.44	1.5E-10	Ribosomal protein S20-like
LOC100909913	100909913	6,258,264–6,282,760	−6.00	5E-8	Norrin-like
Lilra3	100912499	63,920,332–63,929,449	−5.58	3.64E-6	Leukocyte immunoglobulin-like receptor, subfamily A (without TM domain), member 3
LOC100909474	100909474	7–1,408	−5.28	4.16E-05	Protein FAM43A-like
Dgcr2	360742	87,242,441–87,293,721	−4.74	1.17E-03	DiGeorge syndrome critical region gene 2
LOC103689977	103689977	230,186,170–230,198,555	−4.74	1.19E-03	Caspase-6
LOC100910851	100910851	229,264,277–229,343,054	−4.72	1.30E-03	Serine protease inhibitor Kazal-type 5-like
Rin1	207119	220,335,036–220,342,319	−4.54	3.01E-03	Ras and Rab interactor 1
LOC100361850	100361850	87,740,858–87,745,061	−4.54	3.01E-03	Zinc finger protein 74 (Cos52)-like
F9	24946	143,097,507–143,141,791	−4.28	8.79E-03	Coagulation factor IX
Aqp3	65133	57,423,735–57,429,252	−4.28	8.79E-03	Aquaporin 3
Prss29	287136	14,678,286–14,681,877	−4.04	4.53E-04	Protease, serine, 29
Hoxa11	368057	112,500–116,162	−4.03	7.31E-03	Homeobox A11
Raet1e	292450	1,101,666–1,120,340	−3.96	0.03	Retinoic acid early transcript 1E
LOC103689964	103689964	34,940,424–34,946,009	−3.9	0.03	Vacuolar ATPase assembly integral membrane protein Vma21

Based on the dataset, two genes showed an expression change of more than 10-fold; 1 of these genes, i.e., LOC103693999, was upregulated, and 1of them, i.e., LOC108348064, was downregulated. In addition, 18 genes showed expression changes between 5-and 10-fold, with 10 of them being upregulated and 8 of them being downregulated. Detailed information including the top 20 upregulated and top 20 downregulated DeRNAs is listed in Tables 3, 4. Among the DeRNAs that we identified, some are known to be related to inflammation or pain processing, such as IL-1β, zinc finger protein, and TACR1 (NK1). Collectively, the results indicated that pain transition may be associated with the inflammatory response in the DRG.

### 3.3. Real-time quantitative PCR validation of mRNA expression

To validate the reliability of the RNA-Seq results, 6 mRNAs were randomly selected, and their expression was analyzed. Three upregulated mRNAs—helicase (Hells), *N*-acetyltransferase domain containing (Natd1), killer cell lectin like receptor C3 (Klrc3) —and 3 downregulated mRNAs—stimulated by retinoic acid 8 (Stra8), proteoglycan 3 (Prg3), and zona pellucida glycoprotein 2 (Zp2) —were selected. For this experiment, L4–L5 DRG tissues were collected from sham HP model rats and HP model rats 24 h after PGE2 injection. Hells, Natd1 and Klrc3 mRNA expression was significantly upregulated in the HP group compared to the sham HP group (Figures 2C–E). The other three mRNAs were significantly downregulated 24 h after PGE2 injection (Figures 2F–H). The trend of changes in the expression of these mRNAs shown by qPCR was consistent with the RNA-Seq results, supporting the reliability of the RNA-Seq data.

### 3.4. Functional prediction of DeRNAs during pain transition

To explore the molecular mechanism underlying the transition from acute to chronic pain, we further performed GO enrichment and pathway analyses of the DeRNAs between the different groups.

**Figure 3:**
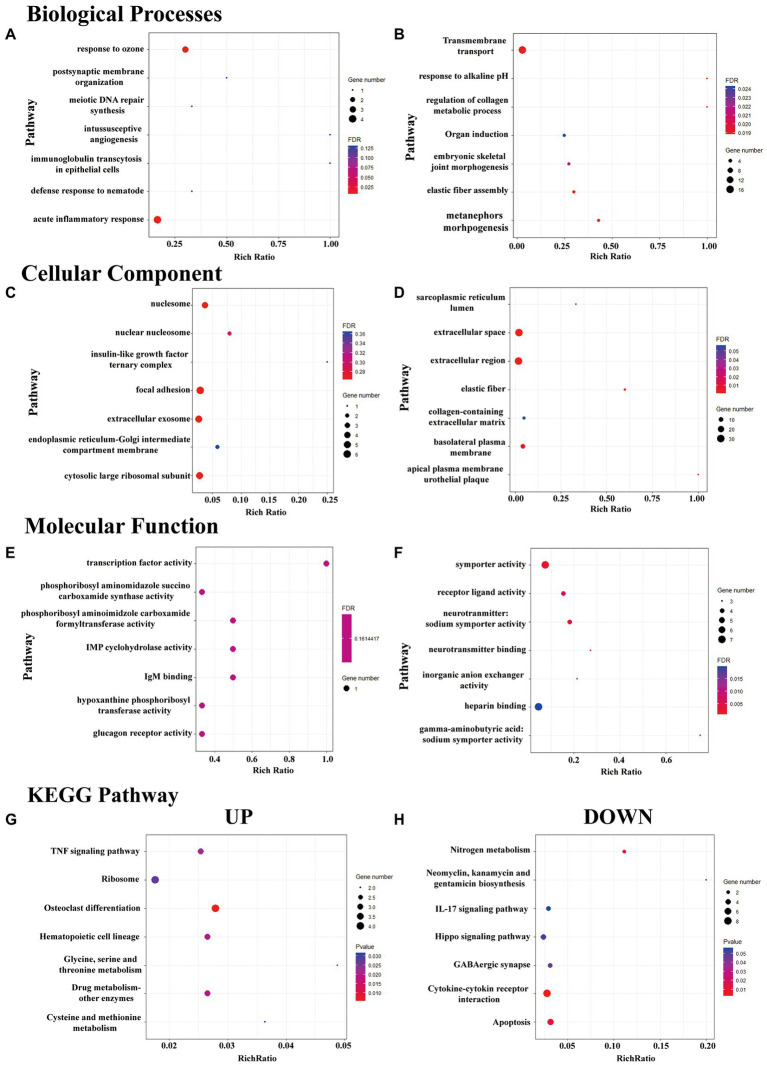
GO enrichment and KEGG pathway analysis of DeRNAs. **(A–F)** The top seven mostly significantly enriched BP, CC, and MF terms for the up- and downregulated DeRNAs. **(G,H)** The top seven mostly significantly enriched pathways for the up- and downregulated DeRNAs.

The BP terms in which the upregulated DeRNA in the HP group were most significantly enriched were acute inflammatory response and response to ozone (Figure 3A). The BP terms in which the downregulated DeRNA in the HP group were most significantly enriched were mesenchymal to epithelial transition involved in metanephros morphogenesis, response to alkaline pH and regulation of collagen metabolic process (Figure 3B).

The CC terms in which the upregulated DeRNA in the HP group were most significantly enriched were nucleosome, focal adhesion, cytosolic large ribosomal subunit and extracellular exosome (Figure 3C). The CC terms in which the downregulated DeRNA in the HP group were most significantly enriched were extracellular space, elastic fiber, and extracellular region (Figure 3D).

The MF terms in which the upregulated DeRNA in the HP group were most significantly enriched were transcription factor activity, IgM binding, IMP cyclohydrolase activity (Figure 3E). The MF terms in which the downregulated DeRNA in the HP group were most significantly enriched were γ-aminobutyric: sodium symporter activity, symporter activity and neurotransmitter: sodium symporter activity (Figure 3F).

KEGG pathway enrichment analysis of the DeRNAs between the sham HP and HP groups was further performed. However, few pathways met the criterion for significant enrichment (FDR < 0.05). Therefore, the top three pathway in which the upregulated and downregulated genes were most significantly enriched were identified. They were glycine, serine and threonine metabolism, drug metabolism – other enzymes, osteoclast differentiation (Figure 3G) and cytokine-cytokine receptor interaction, nitrogen metabolism and apoptosis (Figure 3H).

### 3.5. Identification of CXCL1 in the DRG as a key player in mediating pain transition in the HP model

We hypothesize that identifying overlapping DeDNAs between classical pain models and the HP model might help to identify potential targets for pain treatment. However, few overlapped DeRNA was identified. These DeRNAs were listed in Supplementary Figure S1.

**Figure 4:**
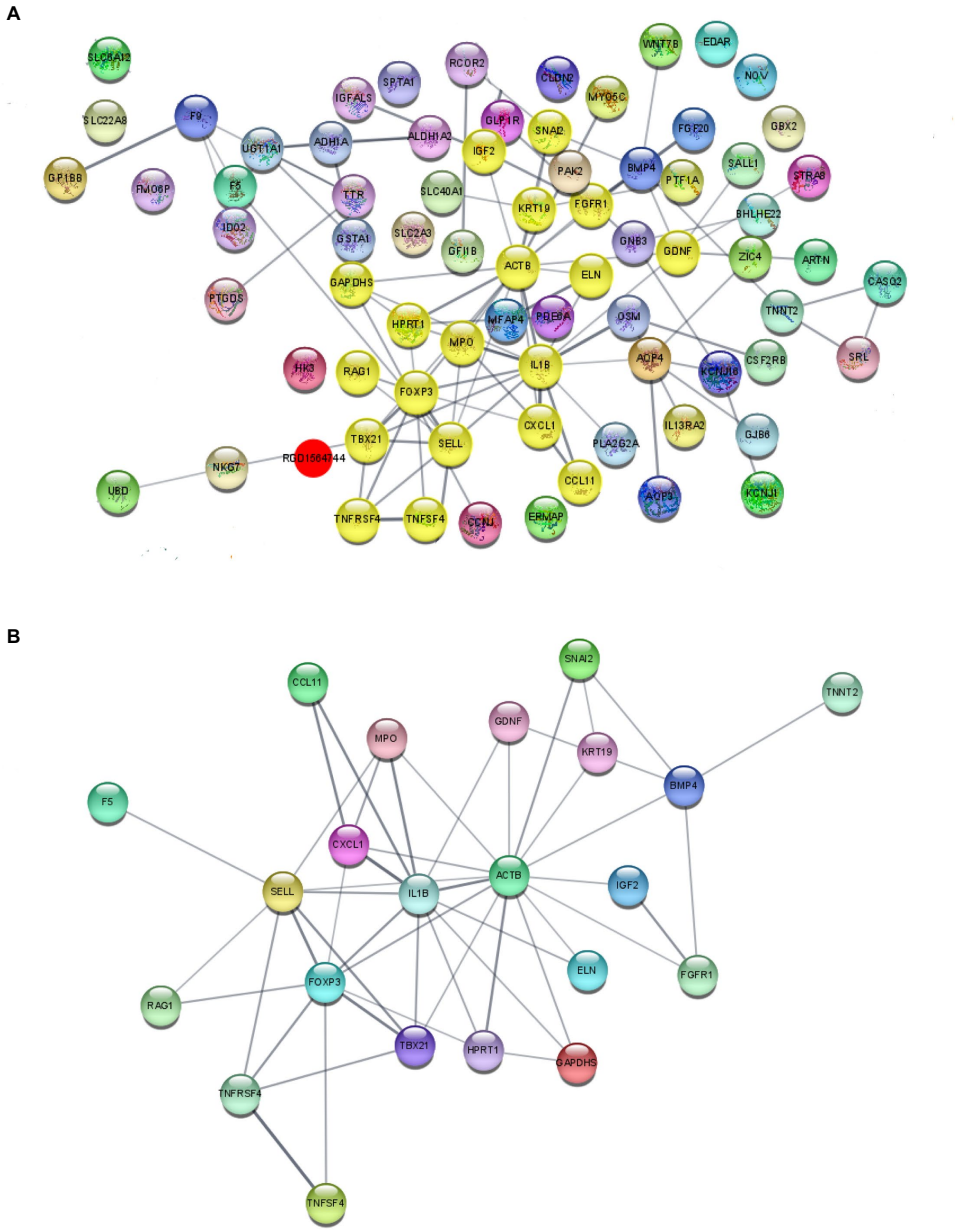
Protein–protein interaction (PPI) network analysis of DeRNAs. **(A)** PPI analysis of DeRNAs between the sham HP and HP groups. **(B)** Major hub genes identified in the network by using the MCODE tools.

**Figure 5:**
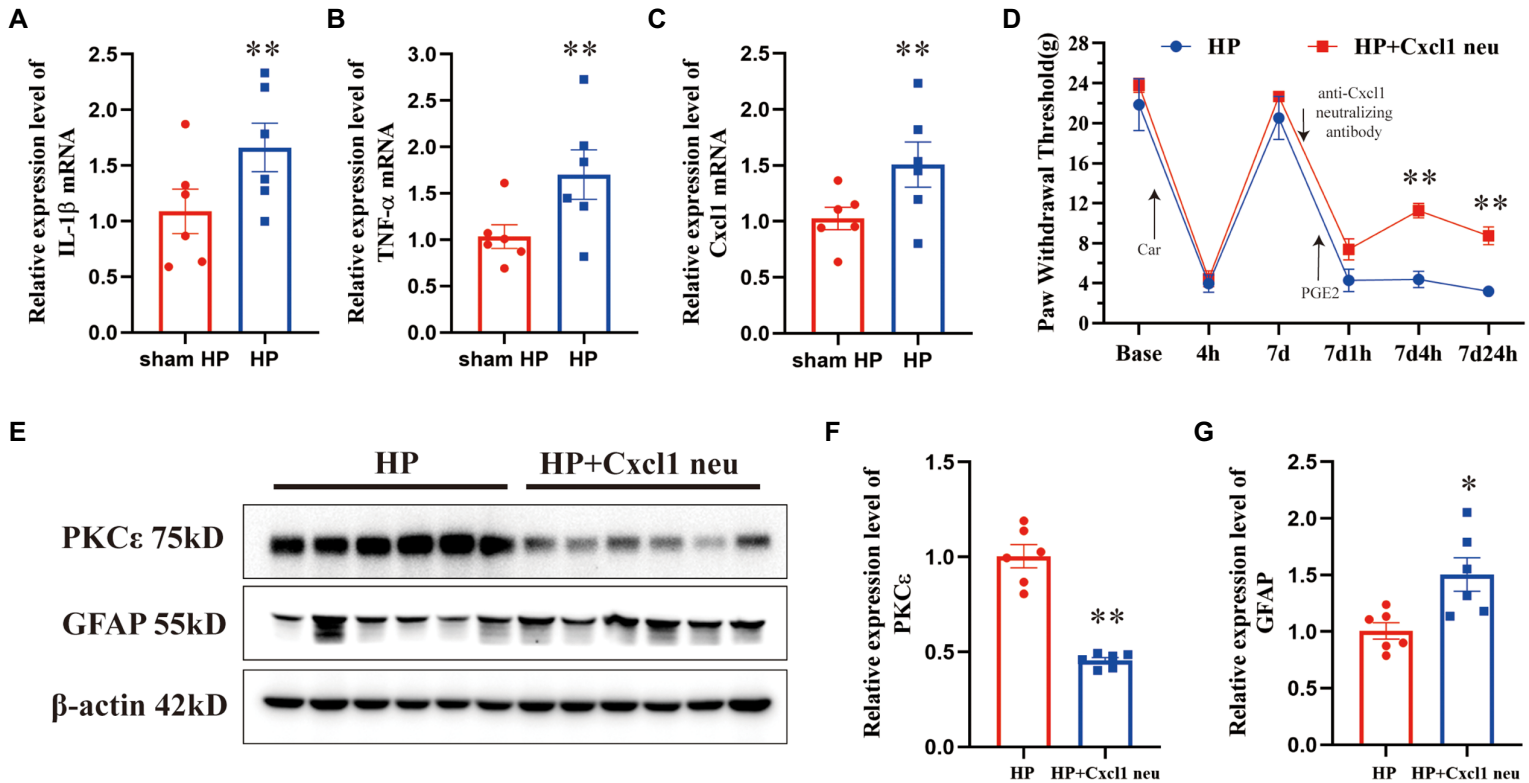
CXCL1 in the ipsilateral DRG plays a pivotal role in pain transition in HP model. The upregulation of IL-1β **(A)**, TNF-α **(B)**, and CXCL1 **(C)** was validated by qPCR. *n* = 6 rats per group. **(D)** Injection of an anti-CXCL1 antibody into the L5 DRG before PGE2 injection increased the pain thresholds of HP model rats 4 and 24 h but not 1 h after PGE2 injection. Representative Western blot showing PKCε **(E, F)** and GFAP **(E, G)** protein expression in the DRG 24 h after PGE2 injection and quantification of the Western blots results. *p < 0.05, **p < 0.01.

We subsequently performed PPI analysis of the genes that were differentially expressed between the sham HP and HP groups (Figure 4A). We found that the expression of neuroinflammation-related genes was significantly different between the two groups. IL1B, TNF SF4, Cxcl1, Xcl1, Gnb3, Gng13, and Foxp3 were identified as major hub genes by PPI analysis, with the MCODE tools (Figure 4B). Because previous studies have demonstrated that IL-1β and TNF-α are widely involved in the interaction of SGCs with neurons in many pain models and contribute to neuroinflammation (Souza et al., 2013; Matsuda et al., 2019), we investigated the mRNA expression levels of IL-1β and TNF-α in the lumbar DRG 24 h after PGE2 injection. The microarray data indicated that the mRNA expression levels of IL-1β and TNF-α were significantly increased 24 h after Car + PGE2 injection, and this finding was confirmed by qPCR (Figures 5A,B). These results are consistent with the above finding that SGC reactivity was induced after Car + PGE2 injection, as previous studies have suggested that IL-1β and TNF-α were mainly secreted by SGCs in the DRG (Matsuda et al., 2019).

CXCL1 contributes to neuropathic and inflammatory pain by mediating neuron–glia interactions in the CNS and PNS (Silva et al., 2017). Therefore, we further tested whether CXCL1 is highly expressed in the DRG 24 h after HP model establishment. The mRNA expression level of CXCL1 was significantly increased 24 h after Car+PGE2 injection (Figure 5C). To further confirm the role of CXCL1 in pain transition, an anti-CXCL1 antibody was directly injected into the L5 DRG. Injection of the anti-CXCL1 antibody into the DRG significantly increased the pain thresholds of HP rats 4 and 24 h, but not 1 h after PGE2 injection (Figure 5D). Furthermore, it also significantly prevented PKCε activation in the L5 DRG 24 h after PGE2 injection (Figures 5E,F). However, anti-CXCL1 antibody injection did not inhibit the activation of SGCs in the L5 DRG (Figures 5E,G). Collectively, these results indicated that CXCL1 is involved in SGC-mediated PKCε activation and pain transition.

## 4. Discussion

Neuroinflammation, a form of inflammation occurring in both the PNS and CNS, has long been considered a key mechanism underlying chronic pain. However, how peripheral neuroinflammation results in chronic pain is still not clear. In this study, we detected a neuroinflammatory process in the DRG involving activation of SGCs and increased release of CXCL1 during the transition from acute to chronic pain. Blocking the function of CXCL1 without inhibiting SGCs activation partially prevented chronic pain. The results indicated that neuroinflammation produced by peripheral nociceptors in the PNS may lead to chronic pain.

It is well established that glial cell overactivation and increased production of cytokines and chemokines are two features of neuroinflammation (Ji et al., 2014). SGCs, which share biological properties with astrocytes in the CNS (Ceruti, 2021), are activated during neuroinflammation in the PNS (Matsuda et al., 2019). Previous studies have reported that nerve injury or persistent inflammation not only causes neuronal changes leading to peripheral sensitization but also results in activation of SGCs in the DRG (Blum et al., 2014; Schulte et al., 2022). It is generally known that SGCs overactivation contributes to peripheral neuroinflammation and pathological pain *via* SGC-neuron interactions. A previous study suggested that the activation level of SGCs in the DRG, which is increased by acute peripheral inflammation, returns to the normal level and that this phenomenon is accompanied by resolution of inflammation (Souza et al., 2013). Moreover, persistent inflammatory pain and neuropathological pain in animals are always accompanied by continued inflammation and nerve injury. All of the above findings suggest that the presence of nociceptive stimuli may be necessary for SGC activation. Here, we observed that the duration of SGC activation in HP model rats was significantly longer than that in rats injected with only PGE2. Because HP model rats had no obvious peripheral inflammation or nerve injury 24 h after PGE2 injection, the result indicates that SGCs remain activated even in the absence of nociceptive stimulation. A function decreases in nitrogen metabolism, which was predicated by KEGG pathway analysis, might be the underlying mechanism of persistent SGC activation, as NO released from peripheral neuron is able to activate SGCs (Hanani and Verkhratsky, 2021).

Many studies have shown that blockade of SGC activation alleviates not only inflammatory pain but also neuropathological pain (Seal, 2016). In the present study, by administering Fc, which reduces SGC activation and function, directly into the DRG (left L5), we showed that selective blockade of SGC activation not only prevented the long-lasting mechanical hyperalgesia (>24 h) induced by PGE2 injection after Car injection but also alleviated acute pain (1 h). SGC activation is able to induce hyperalgesia or allodynia, as previous studies have demonstrated (Ceruti, 2021). A significant decrease in the PWTs was not observed in sham HP model rats 24 h after PGE2 injection, as shown in Figure 1A. The results indicated that PGE2 did not upregulate the expression of GFAP at that timepoint. Furthermore, a previous study demonstrated that the expression level of GFAP is increased by Car injection but returns to the normal level and that this phenomenon is accompanied by resolution of inflammation (Souza et al., 2013). The PWTs of HP model rats returned to normal levels 7 days after Car injection, as we shown in Figure 1A. The results indicated that even if Car activated SGCs, the GFAP expression level returned to normal levels 7 days after injection. PGE2 injection (7 days after Car) significantly increased the expression levels of GFAP, as shown in Figure 1B and the percentage of neurons surrounded by GFAP-positive cells, as shown in Figure 1C. All of the above results indicated that only PGE2 injection may temporarily induce SGC reactivity and that this SGC reactivity cannot be maintained for 24 h. However, PGE2 injection following Car injection was able to induce SGC reactivity for 24 h. Fc injection before PGE2 injection prevented the increase in the expression of GFAP in the DRG. Therefore, we believe that Fc treatment prevent the upregulation of the GFAP expression 24 h after PGE2 injection. PKCε in the DRG is the main mediator of nociceptive sensitization in the PNS, which causes long-lasting hyperalgesia (>24 h) (Joseph et al., 2003; Parada et al., 2003). Fc injection also decreased the expression level of PKCε, as well as GFAP, in the DRG in this study. Our previous research demonstrated that preventing PKCε activation in the lumbar DRG regulates pain transition (Fang et al., 2021). All of the above results indicate that SGC activation is involved in the transition from acute to chronic pain. More interestingly, Fc also alleviated acute pain in HP model rats 1 h after PGE2 injection, suggesting that rapid and brief SGC activation may be involved in PGE2-induced acute inflammatory pain. This is the first clue that PGE2 injection into the hind paw activates lumbar SGCs. The difference in the participation of SGCs in PGE2-induced acute pain and chronic pain is unclear, and we will further study this topic.

Many cytokines and chemokines have been reported to be involved in neuron–glia or glia–neuron interactions. For instance, ATP and CX3CL1 secreted by peripheral neurons activate SGCs through their respective receptors, i.e., P2X7 and CX3CR1, respectively, in mammals with neuropathological and inflammatory pain (Souza et al., 2013; Liu et al., 2018). Proinflammatory cytokines and chemokines, such as IL-1β, TNF-α, and CCL2, are considered the main contributors to SGC-mediated sensitization of peripheral neurons (Berta et al., 2012; Song et al., 2014; Lin et al., 2019). However, the DeRNA in the DRG were significantly different between HP model rats and SNI/CFA models rats (Supplementary Figure S1), and little is known how SGC activation maintains hyperalgesia in the HP models. In the present study, KEGG pathway analysis revealed that the DeRNAs in the DRGs of HP model rats were enriched in several signaling pathways. Among these pathways, the GABAergic synapse pathway first attracted our attention since our previous study demonstrated that decreased expression of GABAAR is involved in pain transition (Wang et al., 2021). Then we found that the cytokine–cytokine receptor interaction and TNF signaling pathways were also enriched. Cytokine and cytokine receptor interactions mainly mediate neuron–glia crosstalk, which contributes to changes in neuronal excitation and function. Through PPI analysis, we found that a large number of proinflammatory cytokines and chemokines were highly expressed. Some of them, such as IL1β, CCL family members and TNF family members, have been shown to be involved in chronic pain. CXCL1 is also a hub gene that was identified by KEGG pathway analysis and PPI analysis. A previous study showed that inhibition of the CXCL1/CXCR1 pathway in the spinal cord and trigeminal ganglion significantly alleviated allodynia and hyperalgesia (Silva et al., 2017). Moreover, the CXCL1 pathway in DRG neurons triggers neutrophil recruitment and subsequent mechanical allodynia (Zhang et al., 2019). Most importantly, a previous study demonstrated that short-term application of CXCL1 is able to modulate the activity of TRPV1+/IB4+ DRG neurons (Deftu et al., 2017, 2018). In addition, PKCε activation in IB4+ DRG neurons and increased TRPV1 expression play an important role in pain transition (Wang et al., 2018; Fang et al., 2021). Therefore, we hypothesized that CXCL1 may be involved in the transition from acute to chronic pain. As shown in the results, we found that the CXCL1 mRNA expression level was increased after PGE2 injection following Car injection, consistent with the genome-wide expression profiling results. Furthermore, blocking the function of CXCL1 in the L5 DRG using a neutralizing antibody partly ameliorated chronic pain in HP model rats and significantly decreased the expression level of PKCε. All of the above results indicated that CXCL1 is involved in the transition from acute to chronic pain.

A previous study indicated that CXCL1 is secreted from astrocytes in the CNS after peripheral nociceptor stimulation (Ni et al., 2019). Intraganglionic injection of an anti-CXCL1 antibody failed to regulate the increase in GFAP expression in the DRG 24 h after PGE2 injection. Therefore, we speculated that CXCL1 was secreted from activated SGCs and induced PKCε activation in the DRG. We also tried to determine the colocalization between the CXCL1 protein and an SGC marker by immunofluorescence staining. However, the antibody failed to detect CXCL1 in DRG sections. This may have been because the CXCL1 expression level was below the detection range of the primary antibody. We will further explore new experimental techniques, such as *in situ* hybridization or the use of more sensitive primary antibodies, to further investigate CXCL1 expression and the mechanism underlying pain transition in the PNS. We did not expect the anti-CXCL1 antibody injection to increase GFAP expression or induce SGC reactivity. However, the blockade of CXCL1 increased GFAP expression. We hypothesize that the DRG injection may affect the expression of GFAP. According to the data presented in Figure 1E, DRG injection did not affect the GFAP expression level. It is still hard to explain why the GFAP expression level was higher in the anti-CXCL1 group than in the HP group, as HP model rats also received DRG injection. The reason for the increase in GFAP expression induced by anti-CXCL1 antibody injection will be further studied.

## 5. Conclusion

The results of this study suggested that SGC reactivity in the L4–L5 DRGs contributes to HP and acute pain. Activated SGCs may partially cause the chronic pain by secreting CXCL1.

## Data availability statement

The original contributions presented in the study are included in the article/Supplementary material, further inquiries can be directed to the corresponding authors.

## Author contributions

JD and JuF: conceptualization. JuF: data curation. JuF, JiF, and JD: funding acquisition. JuF, SW, DX, XS, YL, and BL: investigation. SW, MY, DX, and XH: methodology. JD: writing – original draft. JuF: writing – review and editing. All authors contributed to the article and approved the submitted version.

## Funding

This research was funded by the National Natural Science Foundation of China (grant number 82174490), and the Zhejiang Medical and Health Science and Technology Project Innovative Talent Project (grant number 2021RC098).

## Conflict of interest

The authors declare that the research was conducted in the absence of any commercial or financial relationships that could be construed as a potential conflict of interest.

## Publisher’s note

All claims expressed in this article are solely those of the authors and do not necessarily represent those of their affiliated organizations, or those of the publisher, the editors and the reviewers. Any product that may be evaluated in this article, or claim that may be made by its manufacturer, is not guaranteed or endorsed by the publisher.
