# Supplementary material for: Satellite glial cells drive the transition from acute to chronic pain in a rat model of hyperalgesic priming
Source: Front Mol Neurosci. 2023 Feb 2;16:1089162. doi: 10.3389/fnmol.2023.1089162 (PMC9931746; doi:10.3389/fnmol.2023.1089162)
Supplement: Supplementary file 1 [file Data_Sheet_1.PDF]

Supplementary Material

Satellite glial cells drive the transition from acute to chronic pain in a rat model of hyperalgesic priming

Junying Du, Min Yi, Danning Xi, Sisi Wang, Boyi Liu, Xiaomei Shao, Yi Liang, Xiaofen He, Jianqiao Fang, Junfan Fang

1 Supplementary Figure 1

A

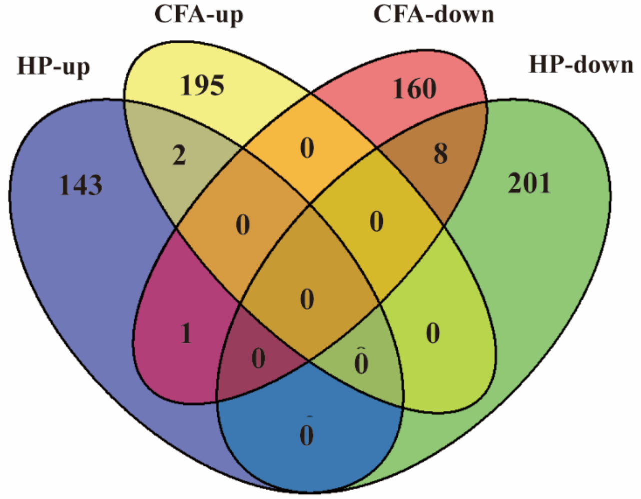

Relationships	Gene symbol	Description
2 common genes in "CFA-up" and "HP-up"	Fgf20	fibroblast growth factor 20
	Tacr1	tachykinin receptor 1
8 common genes in "CFA-down" and "HP-down"	Dnase1l3	deoxyribonuclease 1-like 3
	Slc4a1	solute carrier family 4 (anion exchanger), member 1
	Bmp4	bone morphogenetic protein 4
	LOC259244	alpha-2u globulin PGCL3
	Mfap4	microfibrillar-associated protein 4
	Pi16	peptidase inhibitor 16
	F5	coagulation factor V
	Sall1	spalt-like transcription factor 1
1 common gene in "CFA-down" and "HP-up"	Cpa3	carboxypeptidase A3

B

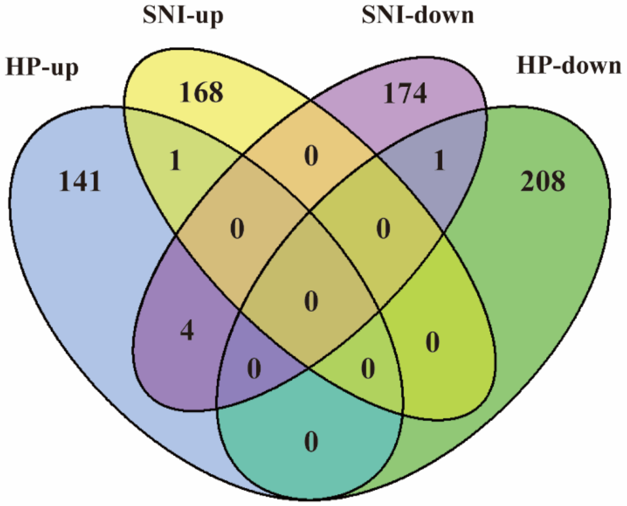

Relationships	Gene symbol	Description
1 common gene in "SNI-up" and "HP-up"	Gsta1	glutathione S-transferase alpha 1
1 common gene in "SNI-down" and "HP-down"	Ttr	transthyretin
4 common genes in "SNI-down" and "HP-up"	Serpina3n	serine (or cysteine) peptidase inhibitor, clade A, member 3N
	Ugt1a1	UDP glucuronosyltransferase family 1 member A1
	Cidea	cell death-inducing DFFA-like effector a
	Rpl30	ribosomal protein L30

Supplementary Figure 1 Comparison of the present HP DeRNA datasets with published DeRNA datasets for inflammatory and neuropathic pain models. (A) Venn diagram showing the overlapping DeRNAs in the DRG between the HP rat model with the CFA rat model. The common DeRNAs are listed in the table on the right. **(B)** Venn diagram showing the overlapping DeRNAs in the DRG between the HP rat model with the SNI rat model. The common DeRNAs are listed in the table on the right.
